# Five functional domains associated with gait performance in Parkinson’s disease and lateral trunk flexion

**DOI:** 10.3389/fneur.2025.1541970

**Published:** 2025-04-08

**Authors:** Keisuke Ota, Hiroki Mani, Keita Nochimura, Yuichi Nakashiro, Shinsuke Hamada, Fumio Moriwaka

**Affiliations:** ^1^Department of Rehabilitation, Hokkaido Neurological Hospital, Sapporo, Japan; ^2^Faculty of Welfare and Health Science, Oita University, Oita, Japan; ^3^Department of Rehabilitation, Sapporo Parkinson MS Neurological Clinic, Sapporo, Japan; ^4^Department of Neurology, Hokkaido Neurological Hospital, Sapporo, Japan

**Keywords:** Parkinson’s disease, lateral trunk flexion, subjective visual vertical, gait performance, gait variability

## Abstract

**Background:**

Lateral trunk flexion (LTF) is a common symptom of Parkinson’s disease (PD). The sensory re-weighting system and sensory-motor function are poor in patients with PD and LTF, and this may cause gait impairment. However, the specific characteristics of gait impairment in patients with PD and LTF remain unclear. The aim of this study was to compare the characteristics of the gait functional domains between participants with PD with and without LTF.

**Methods:**

Fifty-eight patients with PD and Hoehn–Yahr grade 2–3 LTF were divided into two groups: the LTF group (*n* = 22) and the No LTF group (*n* = 36). The Movement Disorder Society Unified Parkinson’s Disease Rating Scale (MDS-UPDRS)-Part III score and subjective visual vertical (SVV) angle were measured. The participants walked with a motion sensor on a straight 20 m path at a comfortable speed. Fifteen gait variables (10 gait cycles) were evaluated and categorized into pace, rhythm, asymmetry, variability, and postural control functional domains and were compared between groups.

**Results:**

The LTF angle, SVV angle; MDS-UPDRS-Part III total, rigidity, and axial scores; and the coefficients of variance for step length, step time, and stance time were significantly higher in the LTF group than in the No LTF group. No other significant differences were observed between the groups.

**Conclusion:**

Participants with PD and Hoehn–Yahr severity 2–3 LTF had greater gait variability than those without LTF, but maintained similar pace, rhythm, asymmetry, and postural control domains. Patients with PD and LTF may develop abnormal neural networks causing greater gait variability.

## 1 Introduction

Postural abnormalities are typical symptoms of Parkinson’s disease (PD). Lateral trunk flexion (LTF) causes lower-back pain and poor postural balance (1–3). Furthermore, the mortality rate is higher in patients with PD and LTF than in those without LTF. Therefore, LTF is the main therapeutic target of physical therapy and requires early intervention (4). It has been suggested that LTF is caused by a poor postural control system that maintains postural equilibrium and orientation and requires complex interactions between the motor, sensory, and cognitive systems (1). Previous studies have revealed that LTF is associated with muscle rigidity (5) and vertical perception based on vestibular information, defined as the subjective visual vertical (SVV) angle (6, 7). The SVV angle is also associated with the Movement Disorder Society Unified Parkinson’s Disease Rating Scale (MDS-UPDRS)-Part III and the Hoehn–Yahr Scale (8). Therefore, poor vertical visual perception may contribute to postural instability. Furthermore, the processing of vestibular information, especially on the ipsilateral lateral trunk flexion side, has been shown to be impaired in individuals with LTF relative to individuals without LTF (9). Vestibular and proprioceptive dysfunction worsen with the progression of parkinsonism (10, 11). Finally, the impaired integration of sensory and motor functions, specifically a poor sensory re-weighting system, may contribute to postural instability in patients with PD and LTF (1, 2, 12).

However, the gait impairment features associated with LTF and the factors related to gait impairment have not yet been fully revealed. Lord et al. reported that gait function consists of five functional domains: pace, rhythm, asymmetry, variability, and postural control (13). They also reported that patients with PD and postural and gait impairments have deficits in the pace, variability, and asymmetry domains relative to those of healthy older adults (14, 15). However, they did not divide patients with PD into LTF and No LTF groups. Thus, the characteristics of the five gait functional domains in the LTF group were not reported. Other previous studies have analyzed some variables corresponding to each functional domain: pace (gait speed), rhythm (double-support time), and asymmetry (step-time asymmetry, swing-time asymmetry), but have found no significant differences in any variables between the LTF and No LTF groups (2, 3, 16). Central pattern generators generate limb movement patterns and regulate rhythms (17). Thus, the pace, rhythm, and asymmetry domains are controlled by central pattern generators. Geroin and Tramonti suggested that individuals with PD and LTF sustain a gait control system based on central pattern generators (2, 16). In contrast, the variability domain is thought to require the integration of sensory information and is thus controlled by the central nervous system involving the spinal cord and cerebral cortex (18, 19). Individuals with LTF show significantly worse executive, attentional, and language functions than those without LTF (20). Additionally, individuals with LTF have reduced functional connectivity in the left insula, bilateral supplementary motor area, and right middle frontal gyrus compared with those without LTF (21). There may be cognitive-motor dysfunction and different processes controlling gait through abnormal neural networks in patients with PD and LTF. Thus, individuals with LTF may exhibit greater gait variability and instability.

This study compared the characteristics of the functional gait domains between individuals with PD and LTF and those without LTF. We hypothesized that gait variability and instability would be greater in the LTF group. Clarifying the characteristics of gait disorders in individuals with LTF will deepen our understanding of the pathophysiology of LTF and serve as the basis for prioritizing treatment in functional areas that are prone to decline.

## 2 Materials and methods

This was a cross-sectional study. Sufficient rest periods were provided between assessments to minimize the effects of fatigue.

### 2.1 Subjects

Fifty-eight patients with PD (mean age: 76.1 ± 8.7 years; disease duration: 9.2 years) participated in this study. The PD diagnosis process followed the Parkinson’s disease Clinical Practice Guidelines 2018 and cases were classified as clinically probable PD, which has a sensitivity and specificity of over 80%. Only patients with a confirmed diagnosis were included in the study. The inclusion criteria were as follows: (1) the ability to walk independently for >20 m and (2) a classification of 2 to 3 on the Hoehn–Yahr severity scale. The exclusion criteria were as follows: (1) medication-induced dyskinesia or (2) visual impairment hindering accurate measurement of the SVV angle.

The participants were divided into two groups: the LTF group (*n* = 22; mean age: 74.5 ± 6.7 years, disease duration: 9.3 years), and the No LTF group (*n* = 36; mean age: 76.8 ± 9.4 years, disease duration: 9.1 years) (Table 1). We captured images showing the lateral view of patients with PD with a variable combination of trunk flexion sides and degrees, calculated according to validated software-based methods. ImageJ software^1^ was used to measure the relevant angles. The LTF angle was defined as the angle between a vertical line and a line along with the fifth lumbar spinous process to the seventh cervical spinous process. Patients with PD who exhibited LTF of more than 5 degrees were defined as the LTF group in the upright position (22). After receiving approval from the Ethics Review Committee of the Hokkaido Neurological Hospital (FY2022, No. 4), an opt-out document was published on the hospital’s official website.

**Table 1:** Comparison of attributes between LTF group and Non LTF group.

	LTF group (*n* = 22)	Non LTF group (*n* = 36)	*p* value	Cohen’s d
Age (Years)	74.5 ± 6.5 (66–89)	76.8 ± 9.4 (55–90)	0.85	0.07
Sex (men/ women)	9/13	16/20	0.15	-
Disease duration (years)	9.3 ± 5.9 (1–20)	9.1 ± 4.9 (2–22)	0.84	0.07
Hoehn-Yahr score	2.8 ± 0.4 (2–3)	2.6 ± 0.6 (2–3)	0.12	0.61
LTF angle (°)	7.4 ± 1.3 (5.3–9.8)	2.6 ± 1.3 (0.6–4.9)	**<0.001**	**2.87**
SVV (°)	7.4 ± 1.2 (3.4–11.3)	3.3 ± 2.0 (0–8.0)	**<0.001**	**1.72**
MDS-UPDRS-partIII score	38.3 ± 8.5 (29–66)	27.2 ± 6.1 (20–42)	**<0.001**	**2.27**
Tremor score	5.1 ± 4.5 (0–13)	4.7 ± 3.6 (0–11)	0.93	0.02
Rigidity score	11.1 ± 2.4 (7–15)	5.1 ± 1.9 (3–10)	**<0.001**	**2.64**
Bradykinesia score	16.8 ± 3.1 (13–22)	15.7 ± 5.1 (6–22)	0.05	0.66
Axial score	7.7 ± 2 0.1 (5–17)	4.9 ± 1.7 (2–8)	**<0.001**	**3.35**

LTF, lateral trunk flexion; SVV, Subjective visual vertical; MDS-UPDRS-part III, Movement Disorder Society-Unified Parkinson’s Disease Rating Scale-Part III; results of each group indicate the mean, standard deviation, and range. Bold values denotes a significant difference at *p* < 0.05.

### 2.2 MDS-UPDRS-part III

The MDS-UPDRS-part III total score and sub-scores for each element were measured. The sub-scores were calculated for tremor (items 15–18), rigidity (item 3), bradykinesia (items 2, 4–8, and 14), and axial scores (items 1 and 9–13) (23, 24).

### 2.3 SVV angle

The bucket method was used to assess the SVV angles (25). Each participant was instructed to sit in a chair with a backrest. They were then instructed to maintain their head and body in a vertical position. The examiner placed a bucket in front of each participant’s face. The participants reported when they perceived that the stick presented at the bottom of the bucket was vertical. The SVV angle was defined as the angle between the vertical axis and the stick. The initial stick position deviated 25° from the vertical axis, and the rotational direction was randomly either clockwise or counterclockwise. If lateral bending of the head or any other disturbances occurred, the SVV measurement was repeated. A positive SVV angle was determined to be in the same direction as the lateral flexion of the trunk. The average angles of four trials were calculated.

### 2.4 Gait function

**Figure 1:**
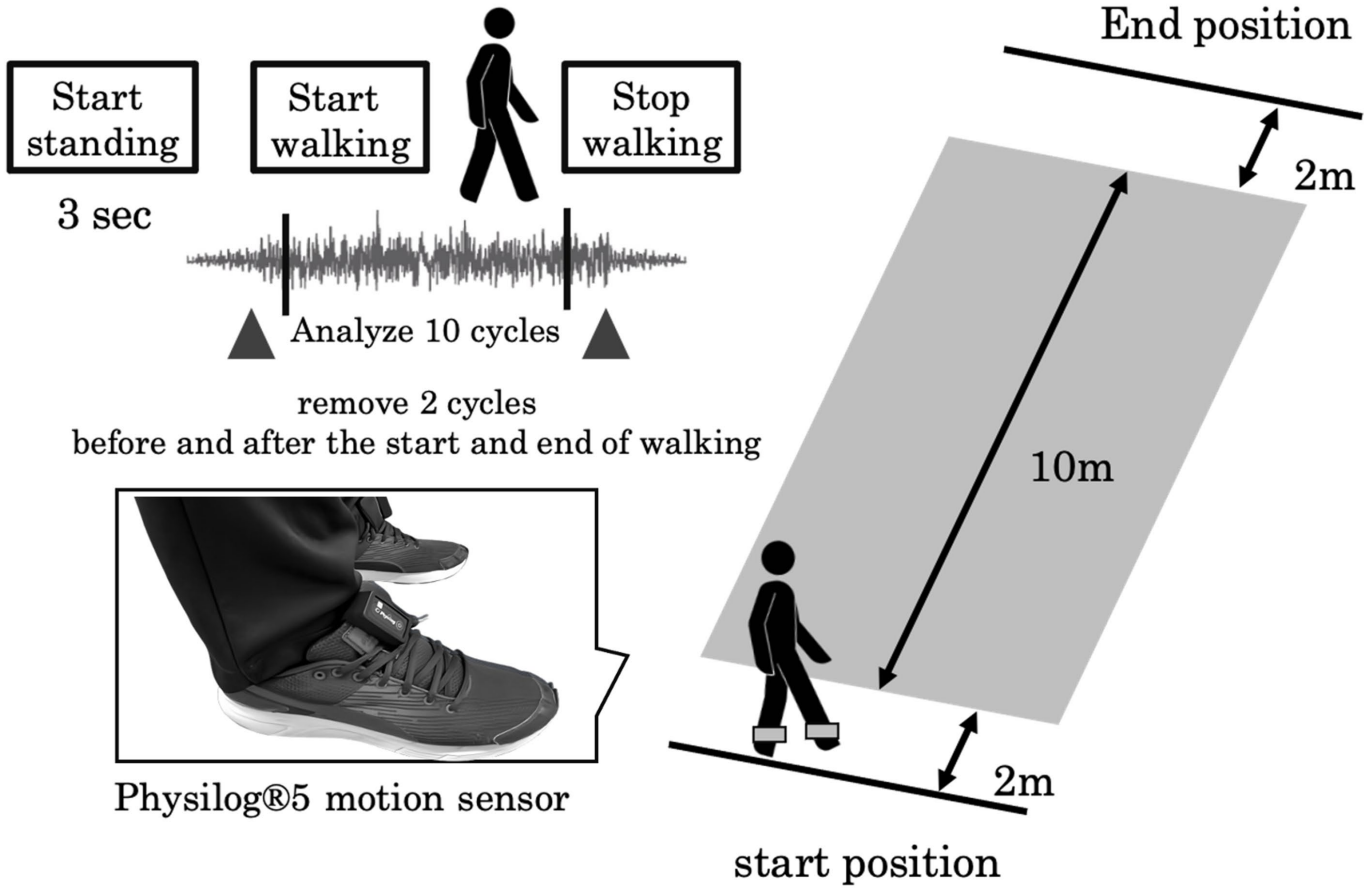
Experimental setup: The participants walked at a self-selected walking speed along a 14 m walkway. A Physilog^®^5 motion sensor was attached to the outside of their shoes.

A six-channel sensory system, Physilog^®^5 (Gait up, Renens, Switzerland; sampling frequency, 128 Hz), was attached to the outside of the participants’ shoes. The participants walked along a straight 14 m path at a comfortable speed. To eliminate the effects of acceleration and deceleration during gait initiation and termination, the analysis range for defining straight gait was the middle 10 m (Figure 1). Data from 10 gait cycles were analyzed. Fifteen gait variables were calculated: the step length (SL), step velocity (SV), step time (ST), stance time (STT), swing time (SWGT), symmetry index of step length (SL_SI), symmetry index of the step velocity (SV_SI), symmetry index of the step time (ST_SI), symmetry index of the stance time (STT_SI), symmetry index of the swing time (SWGT_SI), coefficient of variation in step length (SL_CV), coefficient of variation in step velocity (SV_CV), coefficient of variation in step time (ST_CV), coefficient of variation in stance time (STT_CV), and coefficient of variation in swing time (SWGT_CV). The symmetry index was calculated as previously described (26, 27). These gait variables were categorized into five functional domains according to Lord’s methodology: pace (SL, SV, SWGT_CV), rhythm (ST, STT, SWGT), asymmetry (ST_SI, STT_SI, SWGT_SI), variability (SL_CV, SV_CV, STT_SI, SWGT_SI), and postural control (SL_SI) (13).

### 2.5 Statistical analysis

First, the Shapiro–Wilk test was performed to confirm normality, followed by either an unpaired *t*-test or Mann–Whitney U test, using the Bonferroni correction for between-group comparisons. The categorical variable (gender) was tested using the chi-squared (χ^2^) test. The effect size (Cohen’s d) was calculated. Spearman’s rank correlation coefficient was calculated to assess the association between LTF and SVV angles. JASP was used to perform all the statistical analyses at a significance level of 5%.

## 3 Results

No significant between-group differences were found in age, disease duration, or Hoehn–Yahr scores (*p* = 0.85, *d* = 0.07; *p* = 0.84, *d* = 0.07; *p* = 0.12, *d* = 0.61, respectively; Table 1). Additionally, there was no significant difference in the sex distribution between the groups (*p* = 0.15). The LTF angle; the SVV angle; and MDS-UPDRS-part III total, rigidity, and axial scores were significantly higher in the LTF group than in the No LTF group (*p* < 0.001, *d* = 2.87; *p* < 0.001, *d* = 1.72; *p* < 0.001, *d* = 2.27; *p* < 0.001, *d* = 2.64; *p* < 0.001, *d* = 3.35, respectively; Table 1).

**Figure 2:**
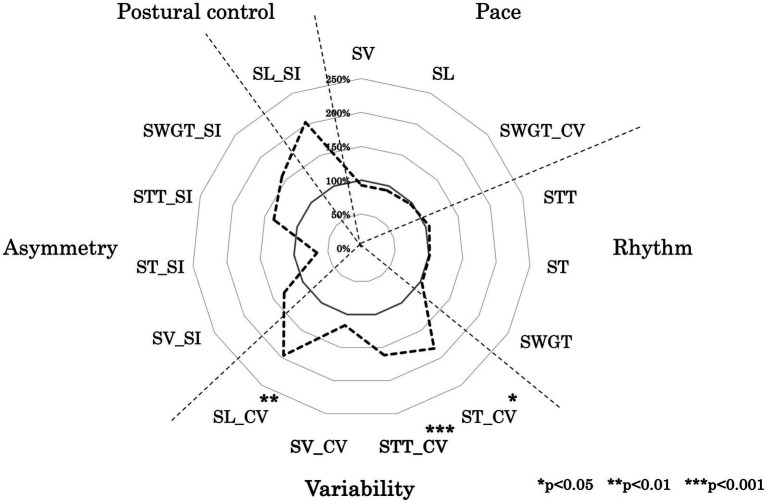
Ladar chart of gait function. This figure illustrates the developmental processes of walking variables in each group, normalized to the average values of the adult group as 100%. The variables included SL (step length), SV (step velocity), SW (step width), ST (step time), STT (stance time), SWGT (swing time), CV (coefficient of variation), and SI (symmetry index).

Figure 2 shows a radar chart illustrating the gait functional domain pattern of the LTF group, with values normalized to those of the No LTF group. Table 2 presents the mean values and standard deviation of the 15 gait variables for each group. ST_CV, STT_CV, and SL_CV were significantly different between groups (*p* < 0.001, *d* = 1.74; *p* < 0.001, *d* = 1.23; *p* < 0.001, *d* = 1.17, respectively; Table 2). No significant differences were observed in the other variables between the groups.

**Table 2:** Spatio-temporal gait parameters.

Gait domain	Spatio-temporal gait parameters	LTF group (*n* = 22)	Non LTF group (*n* = 36)	*P* value	Cohen’s d
Pace	Step velocity (m/s)	123.3 ± 38.6	133.2 ± 41.4	0.52	0.24
	Step length (m)	38.5 ± 13.8	41.5 ± 17.1	0.63	0.18
	Swing time CV	10.1 ± 4.7	10.5 ± 6.3	0.88	0.05
Rhythm	Step time (ms)	3.1 ± 0.8	302.8 ± 53.7	0.51	0.25
	Stance time (ms)	647.8 ± 101.2	639.7 ± 62.6	0.83	0.08
	Swing time (ms)	409.4 ± 62.4	396.7 ± 62.6	0.60	0.20
Variability	Step time CV	8.7 ± 3.6	6.1 ± 2.0	**<0.001**	**1.74**
	Stance time CV	8.8 ± 4.3	4.3 ± 1.9	**<0.001**	**1.23**
	Step velocity CV	9.0 ± 4.0	6.8 ± 3.5	0.29	0.35
	Step length CV	13.7 ± 7.8	7.1 ± 2.9	**<0.001**	**1.17**
Asymmetry	Step velocity asymmetry	3.6 ± 7.6	4.8 ± 7.7	0.70	0.15
	Step time asymmetry	5.3 ± 11.9	3.5 ± 7.7	0.66	0.17
	Stance time asymmetry	1.2 ± 1.0	1.8 ± 1.6	0.42	0.32
	Swing time asymmetry	1.8 ± 1.6	2.8 ± 3.0	0.28	0.44
Postural control	Step length asymmetry	2.5 ± 2.7	5.0 ± 12.0	0.44	0.31

LTF, Lateral trunk flexion; CV, coefficient of variation. Bold values denotes a significant difference at *p* < 0.05.

**Figure 3:**
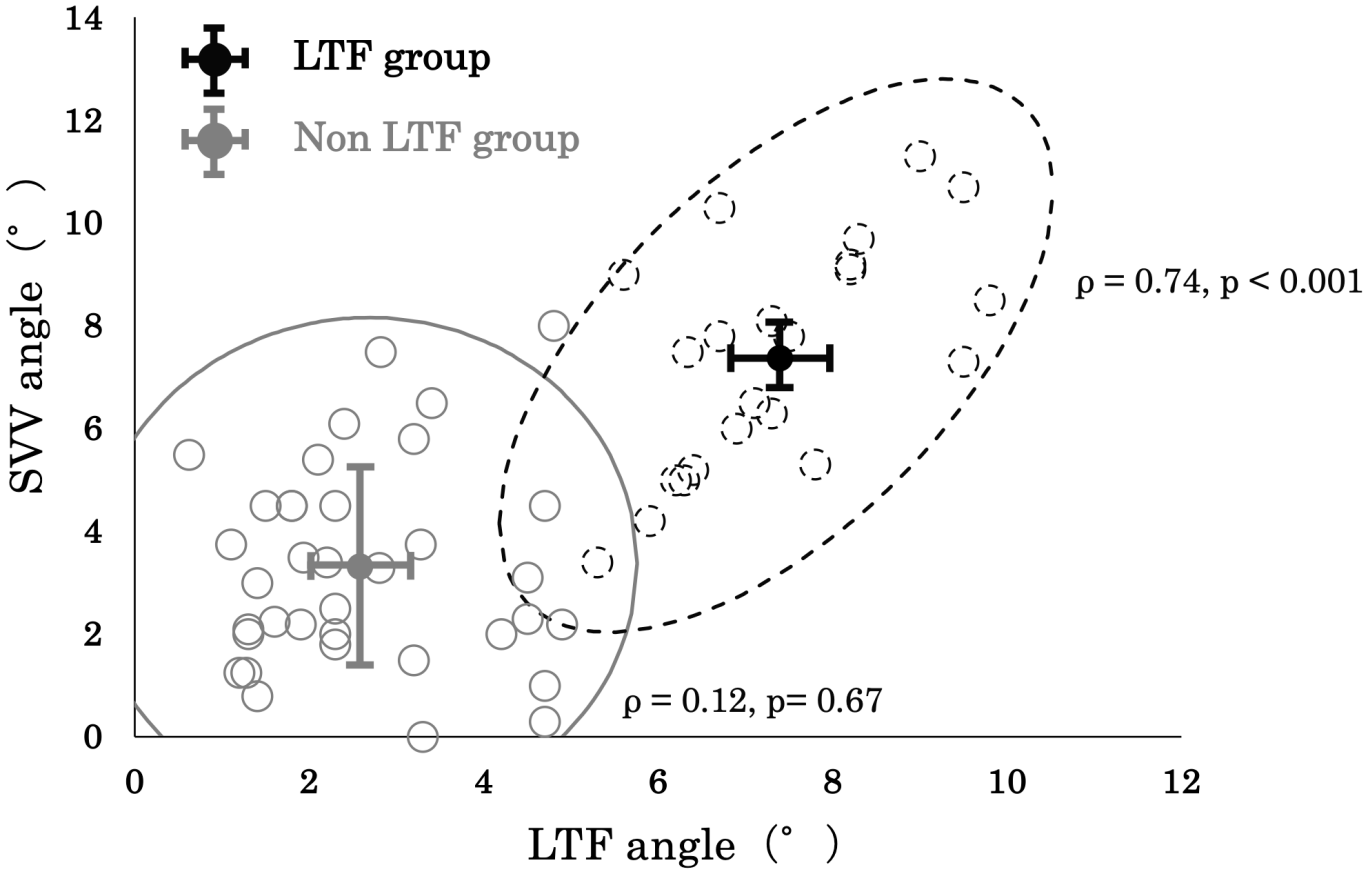
Correlation between the lateral trunk flexion (LTF) angle and the subjective visual vertical (SVV) angle.

Although, no significant correlation between the SVV angle and LTF angle was observed in the No LTF group (*ρ* = 0.12 and *p* = 0.67, respectively), a significantly positive correlation between the SVV angle and LTF angle (*ρ* = 0.74 and *p* < 0.001, respectively) was observed in the LTF group (Figure 3).

## 4 Discussion

This study compared the characteristics of the functional gait domains between participants with PD and LTF and those without LTF. There were no significant differences between the groups in terms of age, disease duration, or Hoehn–Yahr score. Additionally, this study was designed to minimize the influence of fatigue. The LTF group maintained pace, rhythm, asymmetry, and postural control domains, but showed a decline in the variability domain, indicating that gait variability was greater in the LTF group than in the No LTF group.

### 4.1 Variability domain

The LTF group in this study also showed a significantly higher SVV angle, which indicated that they had abnormal vertical perception through the integration of vestibular and visual sensing relative to the vertical perception of those in the No LTF group (Table 1). Gait variability is influenced by the integration of sensory information in the cerebral cortex and spinal cord (18). Gait variability can be regarded as the final motor output based on various types of sensory feedback information (28). Individuals with PD and LTF have been shown to have sensory integration (visual, proprioceptive, and vestibular sensory) disabilities (1, 12) and visuospatial cognitive dysfunction (20, 29) compared to individuals with PD but no LTF. Kohsaka et al. (30) showed that the LTF and SVV angles are associated with hypofunction of the right inferior parietal lobule, superior parietal lobule, and superior temporal gyrus. These regions are the key cortical areas that integrate multisensory signals from the visual, vestibular, and somatosensory systems, which are necessary for postural control and visuospatial cognitive function (17). Additionally, it has been suggested that the control of gait variability is important for the interaction between the cerebral cortex, basal ganglia, and brainstem networks, which integrate motor, sensory, and cognitive systems, respectively (1). However, the interaction between the basal ganglia and cerebellum is disrupted in patients with PD and postural instability (31). The cerebellum is also involved in attentional function (32). There may be a different control process for controlling gait through abnormal neural networks compared with the control process in patients with PD and no LTF. Finally, this study found significantly higher MDS-UPDRS-Part III rigidity and axial scores in the LTF group (Table 1). These physical symptoms may also be related to the observed increase in gait variability (1). Therefore, it is suggested that the LTF group had an increased CV owing to declines in the sensory integration system, visuospatial cognitive function (33), and physical function.

### 4.2 Postural control domain

Contrary to our hypothesis, no significant differences were observed between the groups in the postural control domain. This result was similar to those of previous studies, which also found no group differences in SL_SI (2, 16). Postural stability during walking is achieved through feed-forward and feedback postural control (28). Impaired anticipatory postural control in patients with PD occurs at Hoehn–Yahr stage 2 (34–36). Furthermore, postural reflex impairment in PD appears at Hoehn–Yahr stage 3. Therefore, both groups in this study may have had impaired postural control. Different postural control impairments between the two groups may become prominent from Hoehn–Yahr stage 4 and higher.

Postural sway during gait has been significantly correlated with variables in the pace and rhythm domains (37). Additionally, SL_SI, defined as the postural control domain in this study, may include factors in the pace and asymmetry domains. Therefore, no significant between-group differences were found in the postural control domain, as there were no differences in pace, rhythm, or asymmetry.

### 4.3 Pace, rhythm, and asymmetry domains

There were no significant differences in the pace, rhythm, or asymmetry domains between the groups (Table 2). This is consistent with the findings of previous studies (2, 16) and suggests that the pace, rhythm, and asymmetry domains construct factors associated with basic gait patterns and are controlled by central-pattern generators (17). Variables associated with pace and rhythm, such as walking speed, cadence, and stride, show pronounced impairments from Hoehn–Yahr Stage 4 and beyond (38). Additionally, several studies have reported that asymmetric features disappear at Hoehn–Yahr stage 2 (2, 15, 16). Thus, our results suggest that both groups at Hoehn–Yahr severity stages 2 and 3 may maintain the function of the central-pattern generator.

Parkinsonism progresses from one-sided to bilateral impairment and asymmetry features disappear at Hoehn–Yahr stage 2 (2, 15, 16); however, it should be noted that PD remains an asymmetrical disease. The large standard deviation of the symmetry index value may have hindered the detection of significant differences. The degree of asymmetry varied among individuals with PD, regardless of the presence or absence of LTF (Table 2). Patients with PD at Hoehn–Yahr severity stages 2–3 may exhibit various asymmetrical gait patterns. Therefore, both groups in this study may have had similar bilateral impairments and asymmetrical features could not be detected.

### 4.4 Limitations

This study has several limitations. First, only SL_SI could be calculated for evaluation of the postural control domain because of the limitations of the Physilog®5 system. Lord et al. regarded SL_SI, step width, and the coefficient of variation of the step width as postural control domain values. To identify the detailed characteristics of postural control in individuals with PD and LTF, it will be necessary to include these variables in future studies. Second, although the SVV angle can be measured as an evaluation of vestibular dysfunction, this result involves subjective perception. Therefore, other factors, including cognitive function, may have influenced the SVV angle results. It is important to use objective evaluations, such as qualitative head-impulse tests and eye-movement measurements in conjunction with the SVV angle. Third, the results of this study may only be applicable to individuals with Hoehn–Yahr severity stages 2–3. To identify more detailed gait characteristics in the LTF group, further studies should include subjects with Hoehn–Yahr stages 1 and 4. Fourth, the two groups had a wide range of disease durations (1–22 years). Previous studies have focused on the factors that affect gait variability and found that aging, cognitive function, central nervous system disorders, fatigue, medication, sensory re-weighting ability, and visuospatial cognition have positive effects (28, 39, 40). Disease duration may be a key variable affecting the LTF angle, the SVV angle, and gait control. Further analysis based on disease duration is necessary. Fifth, this study did not assess the UPDRS-Part 2 score. Thus, we were unable to clearly distinguish between tremor-dominant and postural impairment and gait difficulty-dominant types in our patients. Finally, the two groups were divided into 5° LTF angle subgroups; however, subjects with a near threshold were included in both groups. To better understand the characteristics of both groups, further analyses with larger sample sizes and differentiation between subjects near and outside the boundary are necessary.

## 5 Conclusion

This study compared the characteristics of functional gait domains between individuals with PD and LTF and those without LTF. Gait variability was greater in participants with PD and LTF at Hoehn–Yahr stages 2–3 than in those without LTF. In contrast, patients with LTF and Hoehn–Yahr stages 2–3 maintained the pace, rhythm, asymmetry, and postural control domains. The results of this study suggest that, for patients with PD and LTF, it is crucial for physical therapists to implement therapeutic interventions aimed at enhancing their ability to control gait variability. It may be essential to address abnormal vertical perception and improve physical functions, such as rigidity. Highly challenging balance and gait training (41) and/or rigidity control therapy, such as botulinum toxin treatment, may be important. However, there is insufficient evidence to demonstrate that long-term botulinum toxin treatment improves gait function (42, 43).

## Data Availability

The raw data supporting the conclusions of this article will be made available by the authors, without undue reservation.

## Footnotes

^1^ https://imagej.nih.gov/ij/index.html
